# The serum zinc concentration as a potential biological marker in patients with major depressive disorder

**DOI:** 10.1007/s11011-016-9888-9

**Published:** 2016-08-08

**Authors:** Krzysztof Styczeń, Magdalena Sowa-Kućma, Marcin Siwek, Dominika Dudek, Witold Reczyński, Bernadeta Szewczyk, Paulina Misztak, Roman Topór-Mądry, Włodzimierz Opoka, Gabriel Nowak

**Affiliations:** 1 Department of Affective Disorders, Chair of Psychiatry, Jagiellonian University Medical College, Kopernika 21a, 31-501 Krakow, Poland; 2 Laboratory of Trace Elements Neurobiology, Institute of Pharmacology PAS, Smetna 12, 31-343 Krakow, Poland; 3 Department of Analytical Chemistry, University of Science and Technology, Mickiewicza 30, 30-059 Krakow, Poland; 4 Department of Pharmacobiology, Jagiellonian University Medical College, Medyczna 9, 30-688 Krakow, Poland; 5 Department of Inorganic and Analytical Chemistry, Jagiellonian University Medical College, Medyczna 9, 30-688 Krakow, Poland; 6 Department of Epidemiology and Population Studies, Institute of Public Health, Jagiellonian University Medical College, Grzegórzecka 20, 31-531 Krakow, Poland

**Keywords:** Zinc, Depression, Biomarkers, Affective disorders, MDD

## Abstract

Despite many clinical trials assessing the role of zinc in major depressive disorder (MDD), the conclusions still remain ambiguous. The aim of the present clinical study was to determine and comparison the zinc concentration in the blood of MDD patients (active stage or remission) and healthy volunteers (controls), as well as to discuss its potential clinical usefulness as a biomarker of the disease. In this study 69 patients with current depressive episode, 45 patients in remission and 50 controls were enrolled. The zinc concentration was measured by electrothermal atomic absorption spectrometry (ET AAS). The obtained results revealed, that the zinc concentration in depressed phase were statistically lower than in the healthy volunteers [0.89 vs. 1.06 mg/L, respectively], while the zinc level in patients achieve remission was not significantly different from the controls [1.07 vs. 1.06 mg/L, respectively]. Additionally, among the patients achieve remission a significant differences in zinc concentration between group with and without presence of drug-resistance in the previous episode of depression were observed. Also, patients in remission demonstrated correlation between zinc level and the average number of depressive episodes in the last year. Serum zinc concentration was not dependent on atypical features of depression, presence of psychotic symptoms or melancholic syndrome, age, age of onset or duration of disease, number of episodes in the life time, duration of the episode/remission and severity of depression measured by the Hamilton Rating Scale for Depression (HDRS), and the Montgomery-Asberg Depression Rating Scale (MADRS). Concluding, our findings confirm the correlation between zinc deficit present in the depressive episode, and are consistent with the majority of previous studies. These results may also indicate that serum zinc concentration might be considered as a potential biological marker of MDD.

## Introduction

Zinc is a crucial element of living organisms, which is involved in many basic physiological processes (mainly as a cofactor of over 300 enzymes). This cation is recognized as being important for: transcription, translation, DNA repair, proliferation and maturation of cells, apoptosis, neurogenesis, synaptogenesis, neuron growth, or keeping the balance of oxidative and nitrosative redox potential [see (Jurowski et al. 2014 and Szewczyk et al. 2011) for review]. Zinc ions also play a role in the regulation of immune system and inflammation processes, influencing the level of some inflammatory cytokines (e.g. interleukin 6, IL-6; tumor necrosis factor α, TNFα; or interleukin 1β, IL-1β) (Szewczyk et al. 2011; Gapys et al. 2014; Maes et al. 1994, 1997, 1999; Maret and Sandstead 2006; Maserejian et al. 2012; Ranjbar et al. 2013; Russo 2011). It has also been shown that Zn concentration declines during the inflammatory state (Bonaventura et al. 2015).

When compared with other organs, the brain presumably contains the highest levels of zinc in the body. Within brain, Zn^2+^ is nonuniformly distributed and its most abundant in the hippocampus, amygdala, cerebral cortex, and olfactory bulbs (Frederickson 1989, Frederickson et al. 2000; Paoletti et al. 2009). There is evidence that zinc deficiency leads to changes in the central nervous system (CNS) functioning, especially in the glutamatergic transmission in the limbic system and cerebral cortex, which playing an important role in the etiopathogenesis of depression [see (Mlyniec 2015) for review]. Zinc ions can modulate a number of ligand- and voltage-gated ion channels, such as gamma-aminobutyric acid (GABA_A_) (Frederickson 1989; Marchetti 2014), N-methyl-D-aspartate (NMDA) (Chen et al. 1997; Szewczyk et al. 2012), α-amino-3-hydroxy-5-methyl-4-isoxazolepropionic acid (AMPA) and kainate (KA) receptors, and may also affect the serotonin receptors (Kalappa et al. 2015; Veran et al. 2012; Satała et al. 2015). By influencing those receptors in the specific brain areas Zn^2+^ indirectly modulates synaptic plasticity, regulate neuronal signal transduction, and other processes, like memory and learning (Paoletti et al. 2009; Yu et al. 2013; Grabrucker 2014). Furthermore, zinc affects emotional lability, psychomotor functions, attention (Szewczyk et al. 2011; Swardfager et al. 2013a), irritability (Russo 2011) and other emotional, executive and cognitive functions (Szewczyk et al. 2011; Maes et al. 1994, 1997; Swardfager et al. 2013a; McLoughlin and Hodge 1990; Narang et al. 1991; Salari et al. 2015).

Numerous clinical studies confirm the role of zinc deficiency in the development of depressive symptoms in a course of major depressive disorder (MDD) (Szewczyk et al. 2011; Gapys et al. 2014; Maes et al. 1994, 1997; Russo 2011; Swardfager et al. 2013a, b; McLoughlin and Hodge 1990; Narang et al. 1991; Gronli et al. 2013; Siwek et al. 2010; Nguyen et al. 2009; Schlegel-Zawadzka et al. 2000; Siwek et al. 2013). Some of these show correlation between zinc deficiency and drug-resistance in patients (Ranjbar et al. 2013; Russo 2011; Swardfager et al. 2013a). It is also proven, that some antidepressants can influence both the blood and brain zinc level (Siwek et al. 2010; Nowak et al. 2005). On the other hand, several case-control trails confirmed antidepressant activity of zinc supplementation in the depressed patients (Szewczyk et al. 2011; Maserejian et al. 2012; Ranjbar et al. 2013; Lehto et al. 2013; Siwek et al. 2009; Nowak et al. 2003; Salari et al. 2015; LAi et al. 2012).

The aim of this study was to determine the correlation between symptomatology of MDD, and the serum changes of zinc concentration. The presented results are a part of the large clinical study named De-Me-Ter (“Depression – Mechanisms – Therapy”, task 3.2 - Identification of endogenous marker of depression and therapy effectiveness) (Siwek et al. 2016a, b; Siwek et al. 2015; Styczeń et al. 2015).

## Materials and methods

### Recruitment of the study participants

The study participants (Caucasian men and women) were recruited among the in- and outpatients of the Department of Psychiatry, University Hospital, Cracow, Poland, in the period September 21 of 2009 until August 30 of 2013.

Patients fulfilling the DSM-IV-TR criteria for Major Depressive Disorder (MDD) (both in the active phase of depression, and achieve remission) were recruited to the case-control study. All the study participants signed an informed consent and were provided with detailed information (verbally and in writing) about the aims and rules of this clinical study. Each potential study participant had the opportunity to ask questions about the study before singing the consent and all those questions were answered by the doctor responsible for recruitment. The Jagiellonian University Bioethical Committee has approved this study (decision number KBET /77/B/2009; dated June 25, 2009).

The most important exclusion criteria were: diagnosis of a severe psychiatric disorder other than MDD (for example: schizophrenia, schizoaffective disorder, bipolar disorder), substance use disorders (excluding addiction to nicotine and caffeine), comorbidity of serious physical illness (both acute or chronic), diagnosis of a severe personality disorders, breastfeeding, pregnancy or medication which could significantly interfere with blood zinc concentration. As severe somatic diseases excluding from the study (due to the possibility of statistically significant change in the concentrations of biomarkers examined in the De-Me-Ter study) the authors considered the following: chronic autoimmune and inflammatory diseases, acute inflammatory or infections present within a month prior to the recruitment in the study, primary adrenocortical insufficiency, renal failure, chronic pancreatitis, hypoporathyroidism, hyperthyroidism, primary hypoaldosteronism, cancer, megaloblastic anemia due to iron deficiency, thalassemia, hemochromatosis, liver cirrhosis, Wilson’s disease, nephritic syndrome and burns. The additional excluding criteria was the fact of using by the participants the following drugs: hydralazine, nonsteroidal anti-inflammatory drugs (acetylsalicylic acid, ibuprophen, indometacin), tetracyclines, florochinolones, calcium, iron, chelating agents or glucocorticosteroids. All the patients were receiving pharmacotherapy with proven efficacy (mono- or polytherapy), in accordance with the up-to-date treatment guidelines for MDD.

The group of healthy volunteers consisted of people with no present and past history of severe and chronic somatic or psychiatric diseases, without history of substance use disorders (except for caffeine and nicotine abuse), and with no psychiatric disorders in the first-degree relatives. This group was recruited through advertisements on hospital notice boards, by referral from hospital staff, or their relatives and friends.

The detailed socio-demographic and clinical characteristics of the examined population are presented previously (Styczeń et al. 2015).

### The diagnostic tools

For the measurement of the severity of depressive symptoms the Montgomery-Asberg Depression Rating Scale – MADRS (Montgomery and Asberg 1979) and the Hamilton Rating Scale for Depression – HDRS (Hamilton 1960) were used.

### Collection and processing of blood samples. Quantitative analysis of zinc in the blood serum samples

According to the study protocol max. 9.8 ml of blood was obtained from each study participant, at the same time of the day (between 8 and 9 a.m.). Blood was collected from a brachial vein using the Monovette system (Sarstedt, Germany). After the cloth formation, the blood was centrifuged at 1800 xg for 30 min, and the serum (only non-hemolysed) was kept frozen at 80 °C until it could be analyzed. The samples were stored in zinc-free tubes for a maximum period of 4 months. The assessment of zinc was performed in specialized laboratory of trace element analysis, Department of Analytical Chemistry, University of Science and Technology, Cracow. Serum zinc levels were measured by a electrothermal atomic absorption spectrometry (ET AAS) using a PerkinElmer spectrometer model 3110 (USA). The following measurements’ conditions were used: air – acetylene flame, 285.2 nm wavelength, 0.7 nm slit and single-element HCL lamps. Gas flow and burner position were optimized before measurements to achieve high sensitivity. The samples were diluted appropriately to fit into the linear range (1–50 ng/ml) of calibration curves. Standards (Zinc standard solution; Merck Millipore Corp., Darmstadt, Germany) and samples were prepared as water solutions. Diluted samples were homogenized by means of sonification. In the case of samples of extremely low volume (a few microliters) the additive method of sample micro-dilution was used. The lowest concentration traceability for zinc was 0.5 μg/L. Despite dilution, no sample pre-treatment procedures were applied prior to quantitative elements determination. Depending on the total sample volume, triplicate determinations were performed. The accuracy of ET AAS technique was tested by means of recovery analysis, which for Zn was in the range of 94–99 %. All reagents used were of analytical grade. The test tubes for Zn were thoroughly acid washed (0.1 % Nitric acid) and rinsed with double distilled deionized water.

### Statistical methods

The test χ^2^ was used to analyze the differences between the quality variables. The Shapiro-Wilk test was performed in order to evaluate the normal distribution of quantitative data. Because of absence of the normal distribution of data we used the Kruskal-Wallis ANOVA or Mann-Whitney U-test. Correlations between quantitative variables - due to lack of normal distribution - were analysed with the Spearman’s Rank correlation.

## Results

One-hundred-and-fourteen patients (including 28 men and 86 women) who met the DSM-IV-TR criteria for MDD (69 patients were in a depressive episode and 45 were in a remission) were enrolled into the De-Me-Ter case-control study. Among the recruited group of participants there were patients using Selective Serotonin Reuptake Inhibitors (SSRI; 63 patients), Serotonin-Norepinephrine Reuptake Inhibitors (SNRI; 34 patients), tricyclic antidepressants (TCA; 15 patients), mirtazapine (5 patients) and also taking atypical antipsychotic drugs (olanzapine or quetiapine; 15 patients), lithium and lamotrygine (a total of 5 patients) due to enhance the antidepressant therapy (Styczeń et al. 2015). The control group consisted of 50 healthy volunteers (including 14 men and 36 women).

The mean age in the group of patients (49.4 ± 10.7 years) did not show significant differences from the control group (45.8 ± 12.4 years), (*p* = 0.064; Mann-Whitney U-test). There were also no statistically significant differences between the sexes in two groups (test χ^2^; *p* = 0.64). The percentage of women in the examined population of patients was 75 %, and in the control group it was 72 %. In the patient group there were no differences in the mean zinc concentrations between women and men subgroups (*p* = 0.91, Mann Whitney U-test) (Styczeń et al. 2015).

The zinc concentration in the serum samples of patients in depressive episode were significantly lower from those obtained in the healthy volunteers group (*p* = 0.003, Mann Whitney U-test). However, there were no statistically significant differences in zinc levels between patients achieve remission and control group (*p* = 0.348, Mann Whitney U-test) or between depressed stage and remission (*p* = 0.096, Mann Whitney U-test) (Table 1).

**Table 1:** Serum zinc concentration [mg/L] in MDD patients, depending on the current phase of the disease (depression or remission), and healthy controls. Data are expressed as mean ± SD or Median [Upper/Lower quartile] (Mann Whitney U-test, *p < 0.01 vs. Control)

	Depression	Remission	Control
Zn concentration [mg/L]	Mean ± SD		
	0.89 ± 0.18*	1.07 ± 0.48	1.06 ± 0.34
	Median [ Upper/Lower quartile]		
	0.88 [1.01/0.78]*	0.97 [1.09/0.77]	0.98 [1.22/0.82]

Among the group of depressed patients there was no statistically significant difference in zinc levels between patients with and without the following clinical features: atypical or psychotic symptoms of depression, melancholic syndrome, or drug-resistance (Table 2). However, among the group of patients in the remission a significant differences in zinc concentration between patients with and without presence of drug-resistance in the previous episode of depression (*p* = 0.035, Mann Whitney U-test) were observed.

**Table 2:** Comparison of zinc serum concentrations [mg/L; mean (± SD) concentration and median (upper/ lower quantile)] in MDD patients with a various clinical picture of depressive episodes

Depression		Mean ± SD	Median [Upper/Lower quartile]	Mann-Whitney U-test *p*
Atypical features	With	0.87 ± 0.16	0.83 [0.88/0.75]	0.17
	Without	0.99 ± 0.37	0.90 [1.02/0.79]	
Melancholic features	With	0.92 ± 0.18	0.88 [1.0/0.8]	0.68
	Without	1.00 ± 0.40	0.88 [1.02/0.72]	
Psychotic syndromes	With	0.77 ± 0.13	0.78 [0.88/0.65]	0.09
	Without	0.98 ± 0.35	0.88 [1.02/0.79]	
Drug-resistant	With	0.90 ± 0.20	0.88 [1.07/0.72]	0.94
	Without	0.89 ± 0.17	0.87 [1.0/0.79]	

Furthermore, our results showed no significant correlations between zinc levels and age of the patients and some clinical features (for all: patients in depressive phase, remission and total group population): duration of the disorder, average number of hospitalizations in the last year, average number of depressive episodes in the last year, number of total hospitalization throughout life, severity of depression measured by HDRS (a total score), or MADRS (a total score or age of onset) or duration of current episode severe depression, or remission. The only significant correlation was obtained between zinc concentration in patients achieve remission and average number of depressive episodes in the last year (Table 3).

**Table 3:** Correlations between serum zinc concentration [mg/L] and selected quantitative clinical features in depression and remission (Spearman’s Rank correlation, *p < 0.05)

Clinical features	Zn [mg/L]		
	MDD (Total)	Depression	Remission
Age	-0.03	-0.026	-0.101
Age of disease onset	-0.03	-0.026	-0.101
Number of episodes in the life	-0.094	-0.081	0.136
The average annual number relapses in the last year	0.161	-0.012	0.355*
The disease duration	0.092	0.236	-0.107
Duration of the episode /remission	-0.209	0.006	-0.231
Total MADRS score	-0.15	0.033	-0.087
Total HDRS score	-0.13	0.022	-0.023

## Discussion

The presented case-control study demonstrated that the serum zinc levels in patients with depressive episode were significantly lower than those obtained in healthy volunteers’ samples. Moreover, there was no statistically significant differences in Zn^2+^ concentration between patients in remission and healthy volunteers group. The average zinc concentration in patients achieve remission almost reach the control level, albeit statistically zinc levels between acute stage of the disease and remission are not different. Additionally, no significant differences between zinc concentration in patients with or without such clinical features as: atypical features of depression, drug resistance, presence of psychotic symptoms or melancholic syndrome were noticed.

Our findings are in a good agreement with most reports, which demonstrated reduced zinc concentration in the blood (serum, plasma) of depressed patients (Maes et al. 1994, 1997, 1999; Russo 2011; McLoughlin and Hodge 1990; Siwek et al. 2010; Schlegel-Zawadzka et al. 2000; Siwek et al. 2013; Roozbeh et al. 2011; Irmisch et al. 2010; Amani et al. 2010; Salimi et al. 2008; Wójcik et al. 2006; Little et al. 1989). However, in some studies blood zinc concentrations did not differ between depressed patients and control healthy volunteers group (Ranjbar et al. 2013; Narang et al. 1991; Gronli et al. 2013; Nguyen et al. 2009; Manser et al. 1989; Salustri et al. 2010).

Probably the first article which indicated a link between zinc and depression in the clinical studies was published in 1983 and demonstrated the case of major depressive disorder with a low serum zinc level (Hansen et al. 1983). Next article appeared in 1989. Little et al. examined the zinc concentration in serum and urine of depressed patients and demonstrated hypozincemia in 9 out of 30 patients with mood disorders, however, this study did not include the control (healthy) group (Little et al. 1989). In the study on 31 Karachi depressed patients, a significantly lower serum zinc level was shown, but only in depressed women (not in men) compared to healthy controls (Manser et al. 1989). In the following year (1990) McLoughlin and Hodge clearly demonstrated hipozincemia in 14 untreated depressed patients, compared with 14 healthy controls (McLoughlin and Hodge 1990).

Our results are similar to this demonstrated by Maes and colleagues (Maes et al. 1994). In the first study on 48 MDD patients they showed that not only the zinc level was significantly lower in patients group (when compared to healthy volunteers), but also that it was negatively correlated with the severity of depressive symptoms (Maes et al. 1994). In two further studies on 31 and 48 patients (respectively) diagnosed with MDD Maes et al. also confirmed previous observations Maes et al. 1997, 1999). Additionally, they observed that zinc level was much lowered in drug-resistant patients (Maes et al. 1997). The our results are also supported by those obtained by Siwek et al. (2010); Salimi et al. (2008) and Amani et al. (2010).

The data concerning pre- and postpartum depression demonstrated relationship between zinc and depression status (Wójcik et al. 2006; Roomruangwong et al. 2016), as well as bipolar depression in which lower zinc level was characteristic for depressive episode (Siwek et al. 2016b; Stanley and Wakwe 2002).

Moreover, growing evidence for the valuable augmentation effects of zinc adjunctive therapy coming from clinical studies. The studies demonstrated by Ranjbar et al. (2013); Russo (2011); Siwek et al. (2009) and Nowak et al. (2003) showed that zinc supplementation significantly reduced depression severity (depression rating scores) and facilitated the outcome in antidepressant therapy especially in the treatment-resistant patients. Also, zinc monotherapy reduced symptoms of depression in patients with coexisting obesity (Solati et al. 2015).

On the other hand, there are several clinical studies that did not confirmed the presented results. The result obtained by Gronli et al. on 100 patients aged over 64 years (62 women, 38 men) with different psychiatric diagnoses showed that the patients without depression were characterized by a higher deficit of zinc (2013). Similarly, Nguyen et al. noticed in their observational study on 369 women that there is no correlation between presence of depressive symptoms and zinc levels (2009). Also some other groups did not demonstrate alterations in the blood serum of depressed patients (Narang et al. 1991; Irmisch et al. 2010; Salustri et al. 2010).

The dissimilarity between the data presented in those reports may origin from the differences in the time of zinc measurement e.g. depression/remission phase, depression diagnosis – treatment resistant/non-resistant, previous/current pharmacotherapy, duration of illness, gender and dietary variability (Swardfager et al. 2013a, b; Siwek et al. 2013; Nowak 2015).

The mechanisms of the antidepressant activity of zinc may be connected with its influence (modulation) of the neuro, immuno and/or oxidative systems [see (Szewczyk et al. 2011; Siwek et al. 2013; Nowak 2015; Maurya et al. 2016; Tyszka-Czochara et al. 2014) for review].

The obtained results from the presented study confirm the correlation between zinc deficit present in the depressive episode, and are consistent with majority of previous studies. The presented results indicate that zinc blood concentration might be a biological marker of MDD. Nevertheless the zinc level in the course of MDD data still remain ambiguous and should be examined on a bigger populations of depressed patients to widen the knowledge on the role of zinc in the pathophysiology of MDD.
